# Altered Nrf2 Signaling Mediates Hypoglycemia-Induced Blood–Brain Barrier Endothelial Dysfunction *In Vitro*

**DOI:** 10.1371/journal.pone.0122358

**Published:** 2015-03-25

**Authors:** Ravi K. Sajja, Kayla N. Green, Luca Cucullo

**Affiliations:** 1 Department of Pharmaceutical Sciences, Texas Tech University Health Sciences Center, Amarillo, Texas, 79106, United States of America; 2 Department of Chemistry, Texas Christian University, Fort Worth, Texas, 76129, United States of America; Hungarian Academy of Sciences, HUNGARY

## Abstract

Hypoglycemia impairs blood-brain barrier (BBB) endothelial function; a major hallmark in the pathogenesis of various CNS disorders. Previously, we have demonstrated that prolonged hypoglycemic exposure down-regulated BBB endothelial NF-E2 related factor-2 (Nrf2) expression; a redox-sensitive transcriptional factor that regulates endothelial function. Here, we sought to determine the functional role of Nrf2 in preserving BBB integrity and molecular mechanisms underlying hypoglycemia-induced Nrf2 down-regulation *in vitro* using human cerebral microvascular endothelial cell line (hCMEC/D3). Cell monolayers were exposed to normal or hypoglycemic (5.5 or 2.2mM D-glucose) media for 3-24h. Pharmacological or gene manipulation (by silencing RNA) approaches were used to investigate specific molecular pathways implicated in hypoglycemia-induced Nrf2 degradation. BBB integrity was assessed by paracellular permeability to labeled dextrans of increasing molecular sizes (4-70kDa). Silencing Nrf2 expression in hCMEC/D3 cells abrogated the expression of claudin-5 and VE-cadherin, while ZO-1 was up-regulated. These effects were paralleled by a decrease in electrical resistance of hCMEC/D3 monolayers and potential increase in permeability to all labeled dextrans. Hypoglycemic exposure (3-24h) led to progressive and sustained down-regulation of Nrf2 (without affecting mRNA) and its target, NQO-1, with a concomitant increase in the cytosolic pool of E3 ubiquitin ligase, Siah2 (but not Keap1). Pretreatment with protease inhibitor MG132, or selective knock-down of Siah2 (but not Keap1) significantly attenuated hypoglycemia-induced Nrf2 destabilization. While hypoglycemic exposure triggered a significant increase in BBB permeability to dextrans, silencing *Siah2* gene abrogated the effects of hypoglycemia and restored BBB integrity. In summary, our data indicate a potential role for Nrf2 signaling in regulating tight junction integrity and maintaining BBB function. Nrf2 suppression by increased Siah2-driven proteasomal degradation mediates hypoglycemia-evoked endothelial dysfunction and loss of BBB integrity. Overall, this study suggests that sustained activation of endothelial Nrf2 signaling could have therapeutic potential to prevent hypoglycemia-induced cerebrovascular dysfunction.

## Introduction

Hypoglycemia is a potential clinical challenge in the management of type 1 and advanced type 2 diabetes mellitus and aggravates the pathophysiology of long-term microvascular complications associated with diabetes [1–3]. Clinical and preclinical studies suggest that iatrogenic or hypoxia-associated hypoglycemic episodes initiate progressive neuronal injury and wide-spread brain damage resulting in cognitive dysfunction and learning deficits [4–6]. In this line, emerging evidence also indicates a detrimental impact of hypoglycemia on cerebrovasculature at the level of blood-brain barrier (BBB), a physical, transport and metabolic interface that guards and maintains the homeostasis of brain microenvironment [7–9]. For example, we previously demonstrated that exposure to acute and sustained hypoglycemic insult promoted endothelial dysfunction, *via* oxidative and inflammatory stress with significant down regulation of tight junction proteins and consequential increase in BBB permeability [10]. Thus, it is proposed that compromised BBB function during hypoglycemia may trigger the pathogenesis of secondary brain injuries.

A growing body of evidence suggests a pathological role for oxidative stress in the etiology of diabetes-related vascular morbidities [1]. In fact, oxidative stress and inflammation have been predominantly linked to hypoglycemia-induced vascular endothelial dysfunction [11–13] and neuronal injuries [5]. For example, Gogitidze Joy and colleagues [12] observed an increased expression of soluble endothelial inflammation markers in circulation following acute hypoglycemic clamp in healthy and type 1 diabetics, which was further supported by recent findings [11]. Importantly, low glucose conditions inhibit the protective physiological effects of flow-induced shear stress on vascular biology and endothelial responses to flow by abundant accumulation of reactive oxygen species [14]. However, the molecular mechanisms underlying hypoglycemia-elicited oxidative stress and BBB endothelial dysfunction are not well understood and thus, require critical investigation for the development of effective therapeutic strategies to prevent cerebrovascular damage [15].

The pleiotropic functional role of Nrf2-based endogenous defense system in protection and adaptation against oxidative stress and inflammation has been extensively studied [15–17]. Nrf2 is a ubiquitously expressed redox-sensitive transcription factor and a master regulator of constitutive or inducible expression of an elaborate network of molecular systems implicated in redox homeostasis *via* anti-oxidant, drug metabolism, anti-inflammatory, detoxification and radical scavenging functions [17]. Conversely, deletion or down-regulation of Nrf2 potentiates cell susceptibility to the toxic effects of pro-oxidant and inflammatory stimuli, including the collapse of cellular bioenergetics [18–21]. In this context, various studies have attempted to determine the important role of Nrf2-mediated activation of endogenous anti-oxidant defense responses in preserving the functional integrity of BBB; thus, preventing cerebrovascular dysfunction associated with various CNS pathologies [20,22–24]. For example, previous findings from Zhao *et al*. [24] suggested that pharmacological activation of Nrf2 signaling post-brain injury significantly restored the loss of tight junctions (TJ) and prevented BBB disruption. Importantly, recent findings by Aliferi *et al*. [25] have highlighted the potential role of sustained cerebrovascular endothelial Nrf2 activation in preventing BBB breakdown and neurological dysfunction following ischemic stroke. Overall, these findings suggest the potential of targeting Nrf2-dependent defense networks as an effective therapeutic strategy to prevent neurovascular dysfunction. Moreover, Nrf2 activators have recently received considerable pharmaceutical interest [15,26].

Recently, we have demonstrated that prolonged hypoglycemia significantly down-regulates Nrf2 expression in BBB endothelium. However, the mechanistic insight into molecular pathways underlying these effects has not been investigated and it is also unknown whether suppression of Nrf2 expression potentiates hypoglycemia-induced loss of BBB structural and functional integrity [10]. Various endogenous mechanisms such as Keap1 (Kelch-like ECH associated protein 1), and more recently, Siah2 (Seven in absentia homolog 2), have been shown to negatively regulate the Nrf2 activation and signaling under homeostatic and stress conditions [17,27,28]. Importantly, these molecular regulators interact and target Nrf2 for ubiquitin-dependent proteasomal degradation in the cytoplasm [28,29], thus, dictating the Nrf2 stability and its response to stress.

Therefore, the objectives of this study were to: i) assess the potential role of Nrf2 signaling in hypoglycemia-induced endothelial dysfunction and BBB permeability and, ii) address the molecular mechanisms underlying Nrf2 down-regulation by hypoglycemia in brain microvascular endothelial cells using hCMEC/D3 cell line [30]. In addition, we preliminarily tested the therapeutic potential of a novel anti-oxidant tetra-aza macrocyclic ligand, L2 (3,6,9,15-tetraazabicyclo [9.3.1]penta-deca-1(15),11,13-trien-13-ol) [31], to prevent hypoglycemia-induced Nrf2 down-regulation. Our results demonstrated that Nrf2 regulates the expression of endothelial tight and adherence junctional proteins critical for BBB integrity and function. In addition, we found that increased expression and cytosolic localization of Siah2 mediates hypoglycemia-induced Nrf2 down-regulation, leading to endothelial dysfunction and potential loss of BBB integrity.

## Methods

### Materials and reagents

Sterile culture ware was purchased from Fisher Scientific (Pittsburgh, PA, USA) while drugs and other molecular biology grade chemicals were obtained from Sigma-Aldrich (St. Louis, MO, USA) or Bio-Rad laboratories (Hercules, CA, USA). Antibodies were obtained from the following sources: Rabbit anti-ZO-1 (#D7D12), anti-VE-cadherin (#D87F2), and goat anti-mouse (#4408S) and anti-rabbit (#4413S) conjugated to Alexa Fluor 488 and 555 from Cell Signaling Technology (Danvers, MA, USA); mouse anti-β actin (#A5441) and anti-Siah2 (S7945) from Sigma-Aldrich; rabbit anti-Nrf2 (#sc-722), mouse anti-NQO1 (#sc-271116), mouse anti-Keap1 (#sc-365626) from Santa Cruz Biotechnology (Santa Cruz, CA, USA); donkey anti-rabbit (#NA934) and sheep anti-mouse (#NA931) HRP-linked antibodies from GE Healthcare (Piscataway, NJ, USA). Fluorescein isothiocyanate (FITC) and Rhodamine B isothiocyanate (RITC)-dextrans were purchased from Sigma-Aldrich, while Cascade Blue-dextran, anti-claudin 5 (#35–2500) and occludin were obtained from Invitrogen (Eugene, OR, USA).

### Cell culture

HCMEC/D3 cell line, derived from microvascular endothelial cells of surgically excised brain tissue of a female epileptic patient [30], was kindly provided by Dr. P.O. Couraud (INSERM, France) at passage 26. This cell line has been used as a reliable *in vitro* model of human BBB for understanding molecular and cellular regulation of BBB integrity [32–34]. Cells (between passages 28–31) were seeded (2 x 10^4^/cm^2^) on collagen-coated sterile two-well chamber slides (fluorescence imaging), 6-well plates (mRNA expression) or 75cm^2^ flasks (protein isolation and western blotting) and cultured in buffered EBM-2 medium with growth supplements and antibiotics at 37°C with 5% CO_2_ exposure [10] and endothelial phenotype was identified by specific markers, such as CD31 and von Willebrand factor [30].

### Treatment

Following an overnight exposure to EBM2 media with 1% serum (without growth factors, referred as treatment media, TM) cells were exposed to freshly prepared glucose-free DMEM containing either 5.5mM (normoglycemic) or 2.2mM (hypoglycemic) D-glucose, as described earlier [10]. This hypoglycemic concentration and length of exposure were chosen based on previously published *in vivo* [35] and *in vitro* [14] studies using comparable low glucose concentrations and exposure times. Notably, rodent models of hypoglycemia show an average of 2.2 mg/dL plasma glucose levels over a course of 12h following insulin injection [36]. Even more severe forms of hypoglycemia (< 1.1 mmol glucose average) have been tested in primates. 5–6 hours of such severe hypoglycemia were required to induce neurological damage [4]. Although these severe forms (both magnitude and duration) of hypoglycemia are rare events they may occur in diabetic patients. Also, we have previously shown a lack of BBB endothelial cytotoxicity following 12h exposure to 2.2mM hypoglycemia [10]. Additional control experiments were performed to validate the osmotic stress-independent effects of the hypoglycemic treatment [10]. Briefly, cells were exposed to glucose–free DMEM media containing normal (5.5mM D-glucose with 4.5mM L-glucose) or hypoglycemic (2.2mM D-glucose with 7.8mM L-glucose) concentrations.

In separate experiments, hCMEC/D3 cells were pretreated with 1–5μM MG132 (carbobenzoxy-Leu-Leu-leucinal), for 3h [27] or 50-250nM of L2, a novel anti-oxidant [31] for 12h prior to and for the duration of normal or hypoglycemic exposure. Drugs were dissolved in DMSO at final concentration < 0.1%.

### Quantitative RT-PCR

Real time qRT-PCR was performed with a slightly modified procedure adapted from [30]. Briefly, total RNA was extracted from hCMEC/D3 cells using the RNeasy plus mini kit with an in-process genomic DNA decontamination (Qiagen Inc, Santa Clarita, CA). RNA quantity and quality in the samples was analyzed by Nanodrop ND-1000. Complimentary DNA (cDNA) was synthesized from 1μg total RNA using the superscript III first strand synthesis system (Life technologies, Carlsbad, CA) in a total reaction volume of 20μL.

Gene expression in samples was determined by qPCR using SYBR green based florescence method [22]. Using gene specific forward and reverse primer pairs [27], 1μL of cDNA was mixed with SYBR select master mix (Life technologies) in a total reaction of 25μL containing 2μL of each primer (10μM). The primer pairs (sequences shown in Table 1) were custom synthesized by Integrative DNA technologies (Coralville, IA, USA). Amplification was performed on Bio-Rad CFX96 Touch Real-Time PCR detection system and the threshold cycle value (C_t_) for each sample was recorded. Target gene expression (based on C_t_) in each sample was normalized against the house keeping gene (β-actin). Template-free and RT negative controls produced negligible signals with C_t_ > 45. The relative expression (fold change) of target gene in cells exposed to hypo vs. normoglycemic conditions was determined by ΔΔC_t_ method.

**Table 1:** Forward and reverse primer sequences (5' -3') used in real time qRT-PCR.

Target gene	Forward	Reverse
Nrf2 (NM_006164)	CGGTATGCAACAGGACATTG	AGGATGCTGCTGAAGGAATC
Siah2 (NM_005067)	ACCTGGCTATGGAGAAGGTG	CACACCGTCATGAATCGAAC
NQO1 (NM_000903)	TGATCGTACTGGCTCACTCA	GTCAGTTGAGGTTCTAAGAC
Keap1 (NM_203500)	CATCCACCCTAAGGTCATGA	GACAGGTTGAAGAACTCCTCC
β-actin (NM_0011013)	CTGGCCCGGACCTGACAGA	GCCGCAGTGGCCATCTCTT

### Gene silencing by siRNA transfection

A method previously followed by Artus *et al*. [37] was adapted with few modifications. Briefly, hCMEC/D3 cells were seeded at a density of 4–5 x 10^4^cells/cm^2^ in 6-well plates. For BBB integrity assessment (mentioned below), cells were plated on the luminal side of Matrigel coated transwell inserts (polyester membrane, 0.4μm pore size) at a seeding density of 60000 (12-well format) or 40000 cells/insert (24-well format) in EBM2 media. Next day, cell culture media were removed and cells were added scramble or target gene specific Silencer Select pre-designed and validated siRNA (#s9491, Nrf2; #s12838, Siah2; #s18981, Keap1; #40424303, negative siRNA) complexed with Lipofectamine RNAiMAX (Invitrogen) or *Trans*IT-X2 (Mirus Bio, Madison, WI) in OptiMEM I reduced serum media without antibiotics (siRNA final concentration = 20nM/well). After 12h of transfection, reduced serum media was replaced with EBM-2 with all supplements and post 72h transfection cell monolayers were treated with normal or hypoglycemic media and target protein expression was analyzed by immunofluorescence (IF) or western blotting.

### Assessment of BBB integrity

HCMEC/D3 cells were cultured on the luminal side of the transwell chamber as mentioned above. Cells were seeded in a volume of 150 or 400μL in the apical chambers of 24 or 12-well Transwell inserts, respectively. Inserts were placed in 24 or 12-well culture plates containing 500 or 1000μL of EBM2 media per well (basal or abluminal compartment). Cell monolayer integrity was assessed by transendothelial electrical resistance (TEER; Ω.cm^2^) and cumulative paracellular permeability (luminal to abluminal flux) to a mixture of fluorescent dextrans of varying sizes added together (FITC- 4kDa, 7.5 mg/mL; Cascade Blue- 10kDa, 5 mg/mL; and RITC- 70kDa, 7.5 mg/mL), as mentioned earlier [10]. Following the addition of dextran mixture to the luminal chambers (20 or 50uL for 24 and 12-well inserts respectively) abluminal samples (50 or 100uL) were collected from 0–30 min and replaced with an equivalent amount of fresh media. Cell-free blank inserts served as controls. Apparent permeability coefficients (Pe; cm/sec) were calculated from slopes of curves (fitted using linear square regression) obtained by plotting volume cleared versus time for all conditions compared with cell-free blanks and expressed as % control [34,38].

### Immunofluorescence

Protein expression/distribution patterns in fixed cells were analyzed by dual labeling procedure mentioned previously [10,39]. Briefly, hCMEC/D3 cells were fixed with 10% buffered formalin (10 min at room temperature). After washing, cells were permeabilized with PBS containing 0.2% triton x-100 and subsequently blocked with 5% goat serum in PBS at room temperature (RT) for 30 min, followed by incubation with rabbit or mouse (1:100–150) primary antibodies overnight at 4°C. After 3 rinses with PBS, cells were incubated for 1h at RT with Alexa Fluor 488 or 555-conjugated secondary antibodies, respectively (1:800). Cells were rinsed with PBS (3 times) and mounted with DAPI in Prolonged Gold Anti-fade reagent (Invitrogen, OR, USA). Slides were cured overnight in dark and images were captured with EVOS digital inverted fluorescence microscope at 40x magnitude. All images were captured under the same exposure, contrast and brightness settings of the microscope depending on the target protein of interest and any post-processing (contrast enhancement) was performed using the same settings across all conditions. Cells incubated with secondary antibodies without prior primary antibody staining served as negative controls.

### Western Blotting

A previously described procedure was followed [10,39]. Briefly, protein content in cell lysates was determined by bicinchoninic acid assay and equal amount of denatured protein (25–30μg) was subjected to SDS-PAGE (4–20% graded gels or 7.5% gels for Nrf2 detection). Bands were electrotransferred to PVDF membranes for 1h at 100V (or 30V, overnight transfer for Nrf2 and claudin-5), blocked with 5% non-fat dry milk in TBS containing 0.1% Tween-20 (TTBS) for 2h and incubated with primary antibodies overnight (dilution range: 1:300–1:500) in blocking buffer. Blots were washed and incubated with HRP-conjugated secondary antibody (1: 8000) for 2h at RT. After washing, protein bands were visualized by enhanced chemiluminescence using LI-COR C-Digit blot scanner and analyzed by Image Studio software with β-actin as a loading control. Band densities were analyzed by LI-COR Image Studio.

### Statistical Analyses

Data were expressed as mean ± SEM and analyzed by one-way ANOVA followed by *post-hoc* multiple comparison tests using GraphPad Prism Software Inc. (La Jolla, CA, USA). Student’s *t-*test (two-tailed, unpaired) was used when appropriate. For statistical significance, *P* value was set to less than 0.05.

## Results

### Nrf2 critically regulates endothelial barrier integrity and cell metabolism

**Fig 1:**
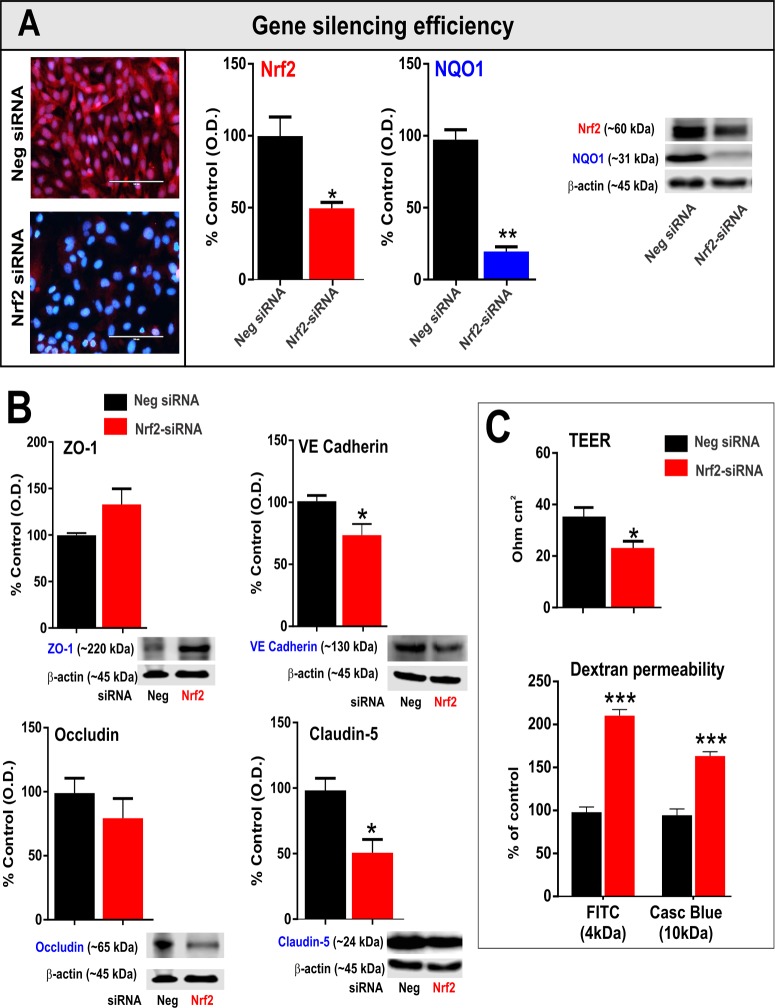
Nrf2 critically regulates BBB endothelial structural integrity and function. **(A)** Nrf2 knock-down efficiency by gene silencing as determined by IF or western blot analyses of Nrf2 (red) and its downstream target, NQO1, in hCMEC/D3 cells transfected with Nrf2-specific or scramble siRNA (n = 3/condition). **(B)** Effects of Nrf2 silencing on endothelial tight and adherence junction protein expression in hCMEC/D3 monolayers. Respective bands with β-actin as loading control were shown at the bottom of the graphs for each protein (n = 3/condition). **(C)** Effects of Nrf2 knock-down on TEER and paracellular permeability to the mixture of labeled dextrans (4 and 10kDa) across confluent hCMEC/D3 monolayers cultured on 12 or 24-well Transwell inserts and transfected with scramble or Nrf2 targeting siRNA (n = 5-6/condition). Images were captured at 40X (scale: 100 μm) and merged with DAPI. Data were expressed as mean ± SEM (% of euglycemic scramble control). ***P < 0.01, **P < 0.01, *P < 0.05, vs. control.

To determine the specific role of Nrf2 in maintenance of BBB integrity, we selectively knocked down Nrf2 expression by siRNA-based gene silencing. As shown in Fig 1A, IF and western blot analyses indicated a significant knockdown of Nrf2 expression in hCMEC/D3 cells after 72h of transfection (compared to scramble control). This finding was also confirmed by profound loss of NQO1 expression, indicating the efficiency of Nrf2 gene silencing and lack of Nrf2 mediated responses [19,21]. For the first time we demonstrate the effects of Nrf2 signaling on BBB endothelial tight and adherence junction protein expression. As illustrated in Fig 1B, western blot analyses indicated that Nrf2 knock-down abrogated the expression of claudin-5 and VE-cadherin in hCMEC/D3 cells (vs. control), without significant effects on occludin expression. On the other hand, ZO-1 was up-regulated in Nrf2 siRNA transfected cells. Notably, the effects of Nrf2 silencing on tight junction proteins are analogous to those observed with hypoglycemia in our earlier study [10].

In line with the altered expression of tight and adherence junction proteins, we observed that abrogation of Nrf2 by siRNA in brain microvascular endothelial cells resulted in a significant loss of BBB integrity, as demonstrated by reduced TEER (by ~38%, Fig 1C) and a parallel increase in paracellular permeability to FITC- (4kDa) and Cascade-Blue (10kDa) dextrans across hCMEC/D3 monolayers, as shown in Fig 1C. Specifically, cumulative permeability (abluminal accumulation, μg/mL) to FITC and Cascade Blue-labeled dextrans increased across the 30 min time frame following the addition of dextrans (*P* < 0.001 for 4 and 10kDa vs. scramble controls; Fig 1C) with the control permeability values being 0.22±0.01 x 10^-3^cm/min for FITC-dextran and 0.13±0.007 x 10^-3^cm/min for Cascade-Blue labeled dextran. It is noteworthy to mention that although hCMEC/D3 endothelial monolayers are not highly impermeable to paracellular passage of low molecular weight solutes (<400Da such as sucrose), they offer better barrier tightness to paracellular tracers of >1000Da, compared to primary endothelial cultures [30,40] and have been used in many in vitro studies for permeability studies [34,37,41].

### Hypoglycemia down-regulates Nrf2 expression and its downstream targets

**Fig 2:**
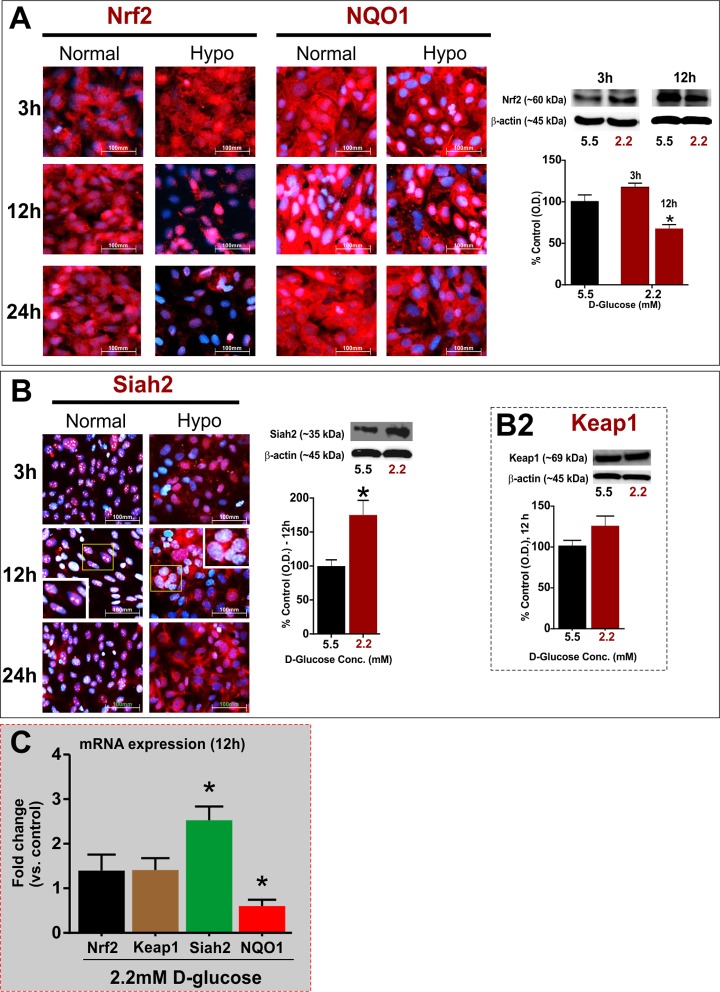
Hypoglycemia induces progressive down-regulation of Nrf2 expression (protein) and function in hCMEC/D3 cells. **(A)** IF and western blot analyses of BBB endothelial Nrf2 and its downstream target, NQO1, expression and distribution following 3-24h exposure to normal or hypoglycemic media (see Methods; n = 3-4/condition). Respective bands with β-actin as loading control were shown above the graphs for each time point. **(B)** Effects of hypoglycemia on protein expression/distribution of intracellular regulators of Nrf2, such as Siah2 (3-12h) and Keap1 (12h; **B2**), as assessed by IF and western blots (n = 3-4/condition). Further, a magnified view of the region represented by yellow box was provided in the inset to demonstrate the cellular localization changes of Siah2 following 12 h exposure to control or hypoglycemic conditions. **(C)** Real-time qRT-PCR based analysis of mRNA expression of target genes in hCMEC/D3 cells exposed to normal or hypoglycemic media (12h) (n = 4/condition). Data were expressed as mean ± SEM (% normalglycemic control for western blots) or fold change over control (mRNA expression). Images were captured at 40X (scale: 100μm) and merged with DAPI. * *P* < 0.05 vs. control. Experiments were repeated twice.

In extension to our previous findings [10], we first determined the longitudinal effects of hypoglycemia on cell lysate Nrf2 expression [28] and its down-stream target, NQO1 [22] abundantly expressed in BBB endothelial cells. As shown by IF staining and western blots (Fig 2A), hypoglycemia caused a significant and progressive down-regulation of Nrf2 and NQO1expression in hCMEC/D3 cells with profound effects at 12-24h (*P* < 0.01 vs. control). However, note that there was an initial increase in the expression and nuclear localization of Nrf2 at 3h post- treatment. For all of the subsequent experiments, we followed 12h exposure time as 24h prolonged exposure could start impacting the endothelial cell viability and may not be clinically feasible. In contrast to the protein expression, one-way ANOVA followed by Dunnet’s *post hoc* analysis indicated that low glucose did not affect steady state Nrf2 mRNA but significantly down-regulated NQO1 mRNA levels (fold-change vs control; F (4,11) = 9.9; *P* < 0.05; Fig 2C). This further confirms the reduced expression/activity of Nrf2 [18], and indicates a post-transcriptional modification of Nrf2 affecting its overall stability [28]. Interestingly, hypoglycemia significantly increased the cellular pool and cytosolic localization of Siah2 with progression of time (3-24h, *P* < 0.05 at 12h; Fig 2B) that correlates with up-regulation of Siah2 transcripts (~2.5 fold increase over control, *P* < 0.01; Fig 2C). Insets shown for 12h Siah2 immunofluorescence represent a magnified view of the region within the yellow colored box for better visualization of Siah2 localization in control vs. treatment conditions (Fig 2B). However, no effects were observed on Keap1 expression either at protein (Fig 2B2) or mRNA levels (Fig 2C).

### MG132 prevents hypoglycemia-induced Nrf2 down-regulation and function

**Fig 3:**
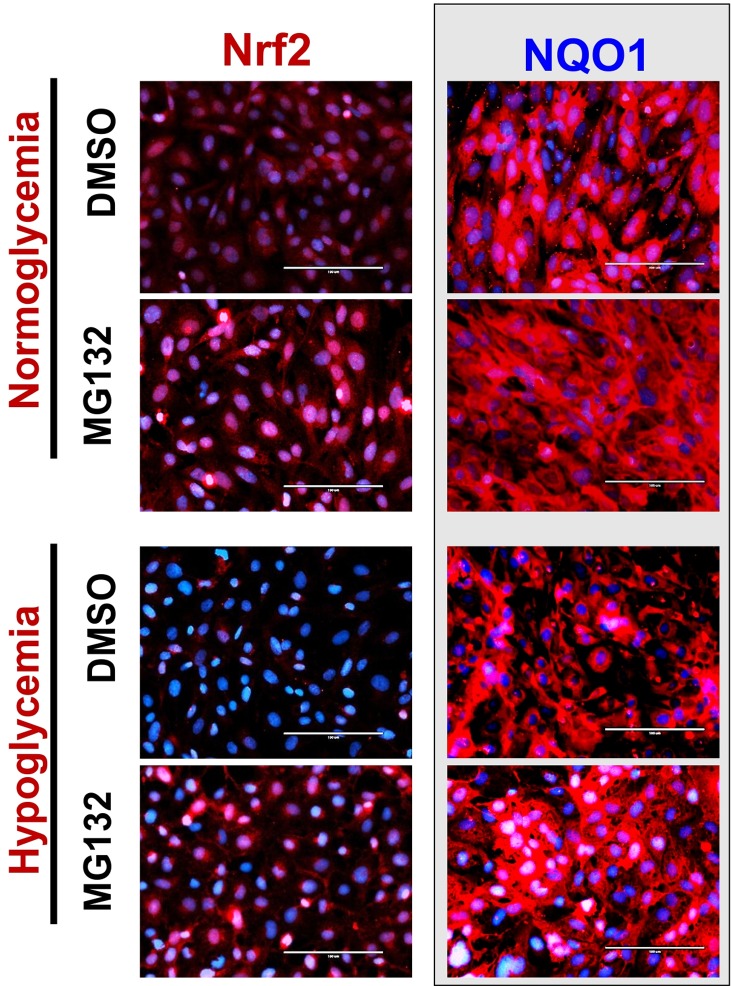
MG132 attenuates hypoglycemia-induced loss of Nrf2 expression and function. HCMEC/D3 cells were pretreated with MG132 (5μM) or DMSO (<0.1%) for 3h and exposed to normal or hypoglycemic media containing MG132 or DMSO (<0.1%) for 12h. Endothelial Nrf2 (red) and NQO1 (red) expression and distribution were assessed by IF analysis with images captured at 40X and merged with DAPI (scale: 100μm). Representative images obtained from two independent experiments.

Given that hypoglycemia up-regulated protein and mRNA levels of Siah2, we next determined the relevant role of Siah2 and proteasome-dependent degradation [42], in hypoglycemia-induced Nrf2 down-regulation. MG132 is a potent inhibitor of ubiquitin-dependent proteasome and has been used previously [27] to block the proteasomal activity downstream to Siah2 function. When tested for the effects of MG132, pretreatment with 5μM concentration attenuated the effects of hypoglycemia and restored Nrf2 expression and function, as assessed by an increase in NQO1 immunoreactivity (Fig 3). It is also noteworthy to mention that MG132 pretreatment alone enhanced Nrf2 expression and nuclear localization, in accordance with recent studies [28]; Fig 3). Overall, these results suggest that increased activation of Siah2-dependent proteasomal degradation mediated hypoglycemia-elicited down-regulation of Nrf2 expression/stability in BBB endothelial cells.

### Siah2 regulates hypoglycemia-induced down-regulation of Nrf2 signaling and loss of barrier integrity

**Fig 4:**
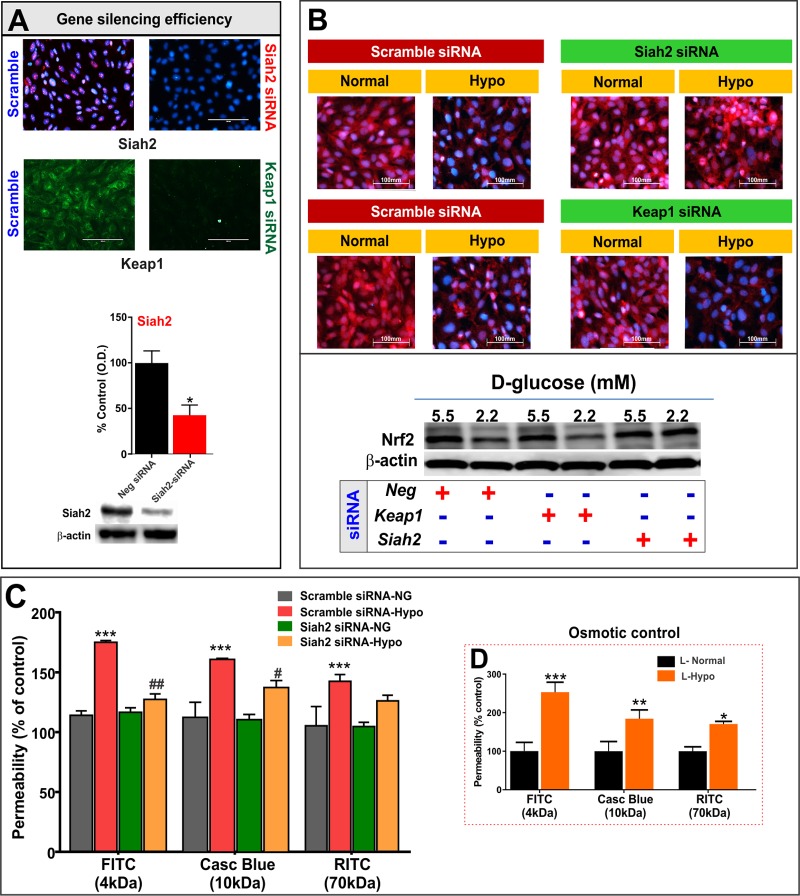
Siah2, but not Keap1, knock-down inhibits hypoglycemia-induced Nrf2 down-regulation and restores endothelial monolayer integrity. **(A)** Gene silencing efficiency and specificity determined by IF staining and western blot analysis of Siah2 (red) and Keap1 (green) in hCMEC/D3 cells transfected with gene specific or scramble siRNA. Respective bands with β-actin as loading control were shown at the bottom of the graph (n = 3/condition). **(B)** IF and western blot analyses of Nrf2 (red) expression/distribution in scramble and Siah2 or Keap1 transfected hCMEC/D3 cells exposed to normal or hypoglycemic conditions (12h) after 72h following transfection (n = 3/condition). **(C)** Paracellular permeability to labeled dextrans of variable size (4-70kDa) across hCMEC/D3 monolayers transfected with scramble or Siah2-specific siRNA and exposed to 12h normal or hypoglycemia following an interval of 72h after transfection (n = 4-6/condition). (**D**) Hypoglycemia-induced increase in dextran permeability is independent of the osmotic effects of the media. HCMEC/D3 cells were exposed to equimolar concentrations of glucose with normalglycemic media (L-normal) containing 5.5mM D-glucose + 4.5mM L-glucose and hypoglycemic media (L-Hypo) containing 2.2mM D-glucose + 7.8mM L-glucose (n = 4-5/condition). Images were captured at 40X (scale: 100 μm) and merged with DAPI. Data were expressed as mean ± SEM (% of scramble control). ***P < 0.001, vs. control; ##*P* < 0.01; #*P <* 0.05, vs. scramble siRNA-Hypo. Experiments were repeated twice.

To further elucidate the role of Siah2-dependent mechanisms underlying the loss of Nrf2 signaling and endothelial barrier dysfunction by hypoglycemia, hCMEC/D3 cells were transfected side-by-side with siRNA targeting Siah2 or Keap1. Cells transfected with scramble siRNA served as control. As revealed by IF and western blot analyses (Fig 4A), siRNA transfection abrogated the protein expression of Siah2 and Keap1 with high efficiency following 72h after transfection.

Importantly, transient knockdown of Siah2 in hCMEC/D3 cells significantly blocked hypoglycemia-induced Nrf2 down-regulation, as demonstrated by IF analysis (Fig 4B, *P>* 0.05, vs. scramble controls). Representative western blots further supported these observations in that no significant differences were observed for Nrf2 expression between normal and hypoglycemic conditions in cells transfected with Siah2 siRNA (Fig 4B), while hypoglycemia down-regulated Nrf2 expression in scramble siRNA controls. By contrast, both IF and western blot analyses revealed that Keap1 knock-down failed to prevent Nrf2 suppression by hypoglycemic exposure (Fig 4B), thus indicating the lack of functional role for Keap1 under these settings. As illustrated in Fig 4C, one-way ANOVA followed by Tukey’s test revealed that paracellular flux of labeled dextrans (4-70kDa) across scramble siRNA-transfected hCMEC/D3 monolayers significantly increased over a 30 min window following hypoglycemic exposure (*P* < 0.001, vs. scramble controls with normoglycemic media) in a size-dependent manner (F(3,14) = 43.7; F(3,14) = 12.09; and F(3,13) = 7.1 for FITC, Cascade Blue and RITC-dextrans, respectively). These results are consistent with the data shown in Fig 4B, and our previous reports. Interestingly, Siah2 knockdown did not affect the dextran permeability under euglycemic conditions; however, transfection with Siah2 siRNA significantly decreased dextran hyper-permeability induced by hypoglycemia (see Fig 4C). For example, Siah2 suppression markedly reduced FITC-dextran accumulation to the control level (*P <* 0.01 vs. hypoglycemic scramble control), while there was a marked reduction in Cascade Blue-dextran (*P* < 0.05 vs. hypoglycemic scramble control). Although, Siah2 knock-down partially inhibited hypoglycemia-induced hyper-permeability to RITC labeled dextran, these effects were not significant.

Also as seen in Fig 4C, the magnitude of permeability reduction by Siah2 siRNA was higher for 4 kDa dextran and followed a size dependent manner. We speculate that siSiah2 could restore the clauin-5 TJ protein expression (that was suppressed by hypoglycemia, [10]) as Siah2 knockdown restores hypoglycemia-induced Nrf2 down-regulation (Figs 4B and 1B). Claudin-5 has been previously shown to regulate a size-selective BBB permeability in that it selectively prevents the permeability of low molecular weight paracellular markers [43,44]. In light of these findings, it is plausible that Siah2 silencing would elicit potential reduction of hypoglycemia-induced permeability to 4kDa dextrans. However, this needs to be further investigated. Overall, these findings reveal a more critical role for Siah2, rather than Keap1, as a molecular regulator of hypoglycemia-induced Nrf2 degradation and BBB endothelial dysfunction.

We also performed osmotic control experiments to determine whether the observed increase in abluminal dextran accumulation following hypoglycemia was mediated by osmotic effects of low-glucose containing media. L-glucose was added to the normal and hypoglycemic medium to make total glucose (D and L-isomers) concentrations equivalent. As shown in Fig 4D, addition of L-glucose to the hypoglycemic media (2.2mM D-glucose) did not affect the increase in paracellular flux of FITC, Cascade Blue or RITC labeled dextrans (*P <* 0.001; *P* < 0.01 and *P* < 0.05; compared to L-glucose added normalglycemic media), which indicated that hypoglycemia-induced loss of BBB endothelial integrity is independent of osmotic effects.

Lastly, we reported a novel mechanism for the macrocycle L2, an anti-oxidant with known potential to counteract transition metal-induced supraphysiological ROS generation and oxidative stress [31]. Our preliminary evidence suggests that L2 significantly induced Nrf2 expression and its downstream molecular target, NQO1 (increased anti-oxidant response) in BBB endothelial cells in a concentration dependent manner (S1A Fig). Given this new role as a potent activator of Nrf2 expression, we next determined if L2 could prevent hypoglycemia-induced Nrf2 down-regulation in hCMEC/D3 cultures. As shown in S1B Fig, pretreatment with L2 (50-250nM) abrogated hypoglycemia-induced suppression of Nrf2 immunoreactivity. The effect was markedly visible even at concentrations that did not affect constitutive Nrf2 expression under control conditions. Thus, these preliminary data provide a novel mechanism for L2 and suggest that this anti-oxidant could prevent hypoglycemia-induced BBB dysfunction by restoring the Nrf2 expression. Nevertheless, additional studies will be needed to further investigate the mechanisms by which L2 activates Nrf2 expression and its potential to prevent hypoglycemia-induced BBB impairment.

## Discussion

Cerebromicrovascular endothelium is highly vulnerable to oxidative/inflammatory stress resulting in BBB dysfunction, via altered composition of intercellular TJ complexes as one of the potential mechanisms [45,46]. Mounting evidence indicates the potential role of Nrf2 in protection and adaptation against oxidative stress and inflammation [17,19,47]. Results from our study strongly implicated a potential neuroprotective role of Nrf2 at the cerebrovascular interface and corroborate previous findings from other groups *in vivo* [20,24,25]. For example, pharmacological activation of Nrf2 with sulforaphane post-injury [24] or primed before experimental stroke [25] increased Nrf2-driven anti-oxidant responses and prevented post-ischemic BBB damage. In addition, Li and colleagues [20] demonstrated that absence of Nrf2 exacerbates brain injury with increased susceptibility to brain edema and BBB breakdown. As suggested by Zhao *et al*. [24] and others [22], regulation of BBB endothelial tight junction protein expression and function could be one of the multifaceted responses triggered by Nrf2 activation. As such, deletion of Nrf2 in the endothelium or progressive loss of Nrf2 during injury [24] could adversely impact the BBB function by impaired cell-cell tight and adherence junction protein expression. Thus, the effects of Nrf2 on tight junction proteins, such as claudin5 and occludin, demonstrated in our study strongly support previous observations with Nrf2 activators [22,24]. Additionally, VE-cadherin was shown to positively regulate the transcription of claudin-5 [48]. Supporting these notions, we have demonstrated that Nrf2 silencing leads to significant down-regulation of VE-cadherin (see Fig 1B). Taken together, our data indicate that increased and persistent levels of oxidative/inflammatory stress by deletion or down-regulation of Nrf2 would negatively impact tight and adherence junction protein expression [49]. This mechanism could compromise the BBB function [8,46] and results in decreased resistance of endothelial barrier.

Herein we show for the first time that Nrf2 regulates BBB endothelial glucose uptake adding to the known repertoire of Nrf2 functions (as shown in Fig 1D). Recent studies have demonstrated that Nrf2 critically regulates the cellular bioenergetics and metabolism by re-directing the metabolic flux through pentose phosphate pathway [21]. Given the high energy demand for a unique structural and functional phenotype of BBB endothelium with abundant glucose transporters and mitochondria [7,9], we speculate that Nrf2-mediated regulation of glucose uptake is vital for BBB function and integrity. Also, recently Muneer and colleagues [50] showed that impairment in endothelial glucose uptake down-regulates tight junction protein expression, thus leading to significant loss of BBB integrity. It is possible that Nrf2 dependent glucose transport in BBB endothelium is critical for the regulation of TJ integrity, but this aspect needs to be further investigated. Hypoglycemia-evoked mitochondrial ROS generation with profound increase in oxidative and inflammatory stress has been shown to cause endothelial dysfunction [12–14] and neuronal injuries [5,6]. Our data strongly corroborates with previously published evidence showing a potential down-regulation of Nrf2 expression in various brain regions of hypoglycemic rats with a concomitant increase in ROS levels [36]. However, in contrast to the findings reported here, Terashima and colleagues demonstrated that low glucose conditions facilitate the induction of Nrf2 expression in HepG2 cells [51]. It should be noted that the low glucose concentration used by these authors was 1g/L, which matches the normoglycemic concentration in our study. Further, it is suggested that Nrf2 responses are cell/tissue-specific based on existing evidence [19], suggesting the differences in Nrf2 response to hypoglycemia between BBB endothelial and hepatocyte cell lines. Thus, it is possible that hypoglycemia-induced Nrf2 down-regulation could be a potential pathway of increased endothelial ROS generation and oxidative stress [13,14], that triggers increased BBB permeability shown in this study and illustrated by others [22,24,25]. Therefore, we propose a more generalized view that hypoglycemia-induced BBB endothelial dysfunction and loss of BBB integrity could be explained by suppression of Nrf2 activity/stability.

Interestingly, hypoglycemia did not affect the mRNA levels of Nrf2 (Fig 2C), thus indicating an influence on post-transcriptional mechanisms underlying Nrf2 down-regulation. A similar response was observed by earlier studies in which hypoxia suppressed Nrf2 protein expression without affecting its transcription [27]. Interestingly, our data also suggested that Siah2 (but not other repressors such as Keap1 [17]) primarily contributed to hypoglycemia-induced suppression of Nrf2 protein in BBB, as knockdown of Keap1 expression did not alter hypoglycemia-induced Nrf2 suppression. Also, the Keap1 expression was relatively unchanged while there was a significant increase in Siah2 mRNA and protein by hypoglycemic exposure for 12h (see Fig 2). Either, deletion of Siah2 or pharmacological blockade of 26S proteasome (a major pathway of Nrf2 degradation, [28,29]) by MG132 restored hypoglycemia-induced Nrf2 function and BBB integrity (see Figs 3 and 4). Under basal conditions, however, Siah2 has no inhibitory effects on Nrf2 expression. In fact, a similar observation was made earlier by Baba and colleagues [27], demonstrating a significant interaction between Nrf2 and Siah2, leading to Nrf2 degradation under hypoxic conditions. In this context, our observations hold significance given the fact that hypoxia is often associated with low glucose conditions during ischemia. Siah2 is an E3-ubiquitin ligase that mediates proteasomal degradation of various target proteins in the cytosol [27,42]. However, results from this study raise interesting questions regarding the possible mechanisms involved in hypoglycemia-induced Siah2 expression and Siah2-Nrf2 interactions, which remain to be investigated further. Nonetheless, our data demonstrate potential role for Siah2-dependent proteasomal degradation in hypoglycemia–induced Nrf2 suppression and loss of BBB integrity.

In conclusion, this study demonstrates the critical role of Nrf2 in maintaining BBB endothelial structure and functional integrity. Our data suggested that acute hypoglycemia significantly impairs the endothelial function and BBB integrity by increased load of oxidative stress due to altered Nrf2 signaling. We provided a mechanistic insight in which increased expression of Siah2 (but not Keap1) by hypoglycemia targets endothelial Nrf2 for proteasomal degradation. Thereby, strategies to inhibit increased Siah2 activity or augment Nrf2 activity could hold therapeutic potential to prevent hypoglycemia-induced cerebrovascular dysfunction.

On a separate note, it is also important to acknowledge limitations inherent to this as any *in vitro* study which provides a somewhat limited picture of the human physiological response to hypoglycemia. Also, as evident from experimental *in vivo* studies, tolerance to hypoglycemic condition (both magnitude and duration) is conditioned by the species used. Although the hypoglycemic conditions simulated in our experiments are quite severe, there are reported cases of rare hypoglycemic crisis in diabetic patients that exceed the experimental parameters tested here (e.g. < 1.1 mmol glucose level for several hours) [4].

## Supporting Information

S1 Fig Effects of novel anti-oxidant and Nrf2 inducer on hypoglycemia-induced Nrf2 down-regulation. HCMEC/D3 cells were pretreated with L2 (50 or 100nM) for 12h and exposed to normal **(A)** or hypoglycemic (**B**) media containing L2 for 12h. Nrf2 (red) and NQO1 (green) expression was analyzed by IF staining with the images captured at 40X (scale: 100μm) and merged with DAPI.
(TIFF)
