# Supplementary figures and images for: Altered Nrf2 Signaling Mediates Hypoglycemia-Induced Blood–Brain Barrier Endothelial Dysfunction In Vitro
Source: PLoS One. 2015 Mar 25;10(3):e0122358. doi: 10.1371/journal.pone.0122358 (PMC4373930; doi:10.1371/journal.pone.0122358)

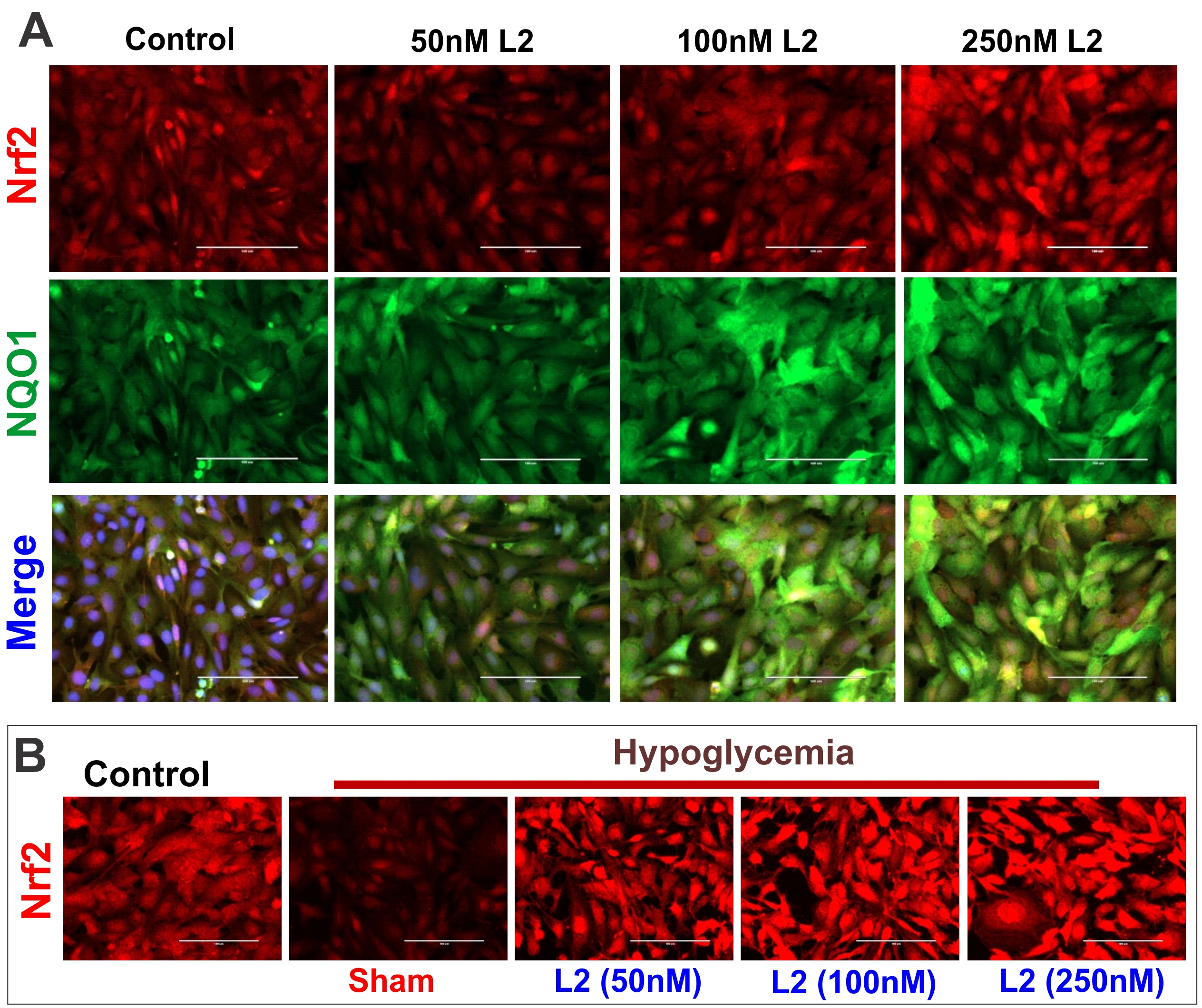

Supplement: S1 Fig — HCMEC/D3 cells were pretreated with L2 (50 or 100nM) for 12h and exposed to normal (A) or hypoglycemic (B) media containing L2 for 12h. Nrf2 (red) and NQO1 (green) expression was analyzed by IF staining with the images captured at 40X (scale: 100μm) and merged with DAPI. (TIFF) [file pone.0122358.s001.tiff]
